# Levels of Mercury, Methylmercury and Selenium in Fish: Insights into Children Food Safety

**DOI:** 10.3390/toxics9020039

**Published:** 2021-02-20

**Authors:** Grazia Barone, Arianna Storelli, Daniela Meleleo, Angela Dambrosio, Rita Garofalo, Antonio Busco, Maria Maddalena Storelli

**Affiliations:** 1 Biosciences, Biotechnlogies and Biopharmaceutical Department, University of Bari “Aldo Moro”, 70010 Valenzano, Bari, Italy; grazia.barone@uniba.it (G.B.); arianna.storelli@uniba.it (A.S.); daniela.meleleo@uniba.it (D.M.); rita.garofalo@uniba.it (R.G.); vitopietro.busco@uniba.it (A.B.); 2 Department of Emergency and Organ Transplant, University of Bari “Aldo Moro”, 70010 Valenzano, Bari, Italy; angela.dambrosio@uniba.it

**Keywords:** children, fish, mercury, methylmercury, selenium, molar ratio, HBV_Se_, RDA, PTWI

## Abstract

Total mercury (THg), methylmercury (MeHg), and selenium (Se) concentrations were measured in various commercially important fish species. The benefit–risk binomial associated with these chemicals was assessed in children through the probability of exceeding the provisional tolerable weekly intakes (PTWIs) of the contaminants and the Se recommended dietary allowance (RDA). The Se:Hg molar ratios, selenium health benefit values (HBV_Se_), and monthly consumption rate limits (CR_mm_) for each species were also calculated. THg and Se were analyzed by atomic absorption spectrophotometer (Shimadzu, Milan, Italy), while MeHg was determined by Trace Ultra gas chromatograph connected with a PolarisQ MS (Thermo Fisher Scientific, Waltham, MA, USA). None of the analyzed fish had Hg levels above the European Community regulatory limits, while most large predators had MeHg levels over the threshold concentration set by US EPA. The estimated weekly intakes of THg and MeHg exceeded in many cases the PTWIs and the Se estimated daily intakes were provided from 0.71% to 2.75% of the RDA. Se:Hg molar ratios above 1 and positive HBV_Se_ index suggested that Se in fish could be enough to alleviate the potential toxic effect of Hg. However, high-risk groups as children should consume fish in moderation because a large consumption pattern, especially of swordfish and tunas, might be of concern for health.

## 1. Introduction

Fish has been acknowledged as a healthy addition to any diet, providing high-quality proteins, vitamins, and numerous other important nutrients [1], thereby leading to fish consumption approvals that also comprise children. Moreover, fish is an excellent source of long-chain polyunsaturated fatty acids (PUFAs), and eicosapentaenoic acid/docosahexaenoic acid (EPA/DHA), whose benefits have been widely recognized in adults and especially in children. This evidence is corroborated by various studies showing that omega-3 supplementation during the first 12 months of life is related to permanent effects on brain structure leading to improved cognition, behavior, and school performance in healthy children [2,3]. Nevertheless, concerns regarding potential harms from exposure to certain chemical pollutants present in fishery products have mitigated the perception of fish as a healthy food. The conflict between risk and benefit deriving from their consumption, although widely discussed, has led to contradictory messages that create a highly complex issue not easy to solve. Toxicologists recommend limiting the consumption, especially of certain fish, while nutritionists recommend eating more oily fish [4]. This debate is strongly fueled by the presence in fish of mercury, which is biologically converted in the aquatic environment, by sulfate-reducing bacteria, into methylmercury a lipophilic organic compound known to be the most poisonous among the mercury compounds. The epidemic called “Minamata disease” is the first experience of severe methylmercury poisoning caused by anthropogenic pollution that emerged mainly among fishermen and their families in the Minamata area in Japan. It comes from the fishery product consumption contaminated with methylmercury released from a chemical plant [5]. This toxin accumulating in vital organs of the human body such as kidneys, liver, and especially the brain, can cause a variety of pathologies, including cardiovascular, renal, reproductive, and neurological disorders [6]. Children are identified as a particularly susceptible population group to the toxic effects of mercury, especially to the risk of neurologic impairment due to greater sensitivity during the early stages of brain development. The neurotoxic effects of methylmercury are, in fact, well documented with several articles of neurobehavioral modifications in children with pre-or early postnatal exposure, including cognitive deficits, effects on motor skills, attention deficit, language competence deficit, and decreased learning and memory abilities [7]. In this picture, it becomes imperative to mention the selenium’s role as natural methylmercury and inorganic Hg antagonist, which through several mechanisms strongly reduces the toxic symptoms that would otherwise accompany high mercury exposures [8,9,10]. On the other hand, almost all marine products are reasonable sources of selenium and, therefore, should offer natural protection against the mercury they also contain. Consequently, when examining health issues related to mercury exposure from seafood, this prominent aspect is of great concern for researchers and should be necessarily considered. Nevertheless, plenty of data on mercury exposure from fish consumption are available, but few have evaluated selenium intake with respect to reducing the harmful effects of mercury [11,12,13,14,15,16,17,18]. Looking at the pre-existing situation in Italy, there is a specific scarcity of data regarding the Hg–Se balance in marine organisms [19,20,21,22], but information on possible positive benefits of selenium on mercury toxicity related to fish consumption in the Italian population is absolutely incomplete [23,24], especially in children despite their great vulnerability [24]. To overcome this lack of information the specific objectives of the present study are (1) to determine the levels of total mercury (THg), methylmercury (MeHg), and selenium (Se) in the muscle tissue of different fish species of considerable economic importance, (2) check if the Hg and MeHg concentrations are compliant with European Union and United State Environmental Protection Agency (EPA) safety standards; (3) calculate Se:Hg molar ratio and the selenium health benefit value (HBV_Se_) index for each fish species, (4) evaluate the health benefit/risk caused by the fish consumption in children, comparing the estimated intakes with Hg and MeHg provisional tolerable weekly intakes (PTWIs) and with Se recommended dietary allowance (RDA), and finally, (5) derive consumption limit recommendations for noncancer health effects (CR_mm_).

## 2. Materials and Methods

### 2.1. Sample Collection

A total of 152 specimens from the Mediterranean area and belonging to different fish species (Table 1) were purchased from May 2019 till July 2019 in the main commercial centers of the Apulian region in southern Italy. The species analyzed included demersal-pelagic and benthic fish. For bluefin tuna, swordfish, and albacore, slices from different specimens (*n* = 15) of about 0.1–0.2 kg of muscle tissue were obtained. After collection, the specimens, separated by species and disposed in polythene bags, were carried to the laboratory. The dissection was operated from the dorsal surface of each animal. Fish identification was carried on the basis of illustrated taxonomic keys [25]. For each species, a composite sample was prepared, homogenized, and stored below −20 °C, pending analysis. All the utensils and containers utilized for handling and dissection (ceramic knife) of samples were conserved in HNO_3_ solution (10%) overnight and rinsed many times with ultrapure water prior to use.

### 2.2. Reagents, Standard, and Reference Material

All solvent used (hexane, toluene, methanol, and acetone) (Carlo Erba, Milan, Italy) were of pesticide analysis grade and all acids used (nitric acid (65%) (Merk, Darmstadt, Germany), hydrochloric acid (37%) (Carlo Erba, Milan, Italy), sulfuric acid (96%) (Carlo Erba, Milan, Italy), and glacial acetic acid (Merk, Darmstadt, Germany) were of analytical reagent grade. The reagents used were NaBH_4_ (Merk, Darmstadt, Germany), cysteine (Fluka, Munchen, Germany), NaBPh_4_ (Merk, Darmstadt, Germany), CuSO_4_ (Merk, Darmstadt, Germany), NaCl (Merk, Darmstadt, Germany), and sodium acetate (Carlo Erba, Milan, Italy). Adequate amounts of deionized water were used to prepare daily solutions of these reagents. THg and Se standard (AppliChem, Darmstadt, Germany) solutions were made by dissolving suitable pure quantities in acidified water (HNO_3_ 0.3%), while methylmercury chloride was obtained from Alfa Aesar, (Heysham, U.K.) (99.4%). Stock solutions were prepared by dispersing apposite quantities of salt in deionized water. Methylmercury chloride (Alfa Aeser, Heysham, U.K.) and ethylmercury chloride (TRC, Toronto, ON, Canada) standard stock solutions were prepared by dissolving the appropriate amounts of pure compounds in methanol. Standard solutions of phenylated organomercury compounds (MeHgPh, EtHgPh) in hexane at different concentrations were prepared by reacting different amounts of the above standard stock solutions with NaBPh_4_ at pH 3. All standard solutions were stored at 4 °C, away from light before use, and the working standard solutions for each individual mercury species were prepared daily. Certified reference materials (CRMs) were Lobster Hepatopancreas (TORT-3) provided by the National Research Council of Canada (Ottawa, Ontario, Canada). Glassware was rinsed with ultra-pure water, decontaminated overnight in 10% (*v*/*v*) nitric acid solution, and rinsed again.

### 2.3. Chemical and Instrumental Analysis

#### 2.3.1. Sample Preparation and Equipment

The extractive analytical procedure and the instrumental conditions to determine total mercury (THg) and selenium (Se) concentrations have been described in detail elsewhere [20]. Briefly, aliquots of samples (about 2 g) were digested to a transparent solution with a mixture of H_2_SO_4_–HNO_3_ (1:1). The sample solution was then cooled and diluted with double distilled water according to the method recommended by the official Italian agencies [32]. THg and Se were analyzed by atomic absorption spectrophotometer (Shimadzu AA 7000, Milan, Italy) equipped with a hydride vapor generator (HVG-1) after reduction by NaBH_4_. For the quantification of organic Hg (MeHg), the protocol described by Ipolyi et al. [33] was followed. Aliquots of the samples (about 0.5 g) were washed with acetone and toluene, consecutively. After centrifugation, the liquid phase was discarded and the sample added of ethylmercury chloride in methanol (100 µL internal standard), and of hydrochloric acid (6 M) was subjected for 30 min to sonication by an ultrasonic bath LBS2 (Levanchimica, Bari, Italy). Subsequently, an aqueous solution of NaCl 10% (*w*/*v*) was added to the sample, and the mixture was centrifuged (2400 rpm for 10 min). The supernatant was extracted twice with toluene and the combined organic extract was subjected twice to back-extraction with a 1% (*v*/*w*) cysteine aqueous solution. After acidification of the collected cysteine extract with H_2_SO_4_ (0.1 M), the derivatization reaction was carried out by adding 1 mL of saturated CuSO_4_ solution and 0.2 mL of 1% (*v*/*w*) NaBPh_4_ aqueous solution in the presence of n-hexane. After 20 min of agitation, the organic phase was separated and analyzed using a Trace Ultra gas chromatograph connected with a PolarisQ MS (Thermo Fisher Scientific, Waltham, MA, USA). A SPB-608 capillary column (30 m × 0.53 mm id., 0.5 μm film thickness) (Supelco, Munich, Germany) was utilized. One μL of the sample was injected in splitless mode at an injection temperature of 250 °C. The transfer line temperature was at 280 °C temperature program, 50 °C × 1 min and then increased at a rate of 20 °C min^−1^ to 280 °C and held for 10 min. Detector temperature was designed at 240 °C. Helium (99.99%) was used as a carrier gas at a flow rate of 1.0 mL min^−1^. Electron impact ionization was performed with an electron energy of 70 eV. A mass range from *m*/*z* 50–350 was recorded in the full-scan mode to check for spectral interferences, while the SIM setup was MeHgPh: *m*/*z* = 292.00, 294.00, and 279.00; EtHgPh: *m*/*z* = 279.00, 306.05, and 308.10. The dwell time was 100 ms. Reporting data were expressed on a wet weight basis.

#### 2.3.2. Quality Control and Assurance

Accuracy and precision were proved by using TORT-3 Lobster Hepatopancreas (National Research Council of Canada). Replicate analyses (*n* = 3) (THg 0.289 ± 0.021 mg kg^−1^ dry weight; MeHg 0.131 ± 0.010 mg kg^−1^ dry weight; Se 11.0 ± 0.98 mg kg^−1^ dry weight) were in accordance with certified values (THg 0.292 ± 0.022 mg kg^−1^ dry weight; MeHg 0.137 ± 0.012 mg kg^−1^ dry weight; Se 10.9 ± 1.0 mg kg^−1^ dry weight), (% recovery = 96–101%). The limits of detection (LOD: 3 SD blank value) and of quantification (LOQs: 10 SD blank value) are the following: LODs: THg: 5 ng g^−1^ wet weight, MeHg: 0.03 ng g^−1^ wet weight, Se: 1 ng g^−1^ wet weight; LOQs: THg 13 ng g^−1^ wet weight, MeHg: 0.12 ng g^−1^ wet weight, Se 3.6 ng g^−1^ wet weight.

### 2.4. Exposure Assessment and Dietary Reference Intake

Estimated daily intakes (EDIs) of Hg, MeHg, and Se through fish consumption in children (age: 3.0–9.9 years old) were determined using the subsequent equation:

EDI = (C × IR)/BW,

where C is element concentration, IR is daily ingestion rate (children: 37.2 g day^−1^), and BW is children’s body weight (26.2 kg) [34]. The resultant values were subsequently compared with the Hg and MeHg toxicological reference intakes, expresses as PTWI (THg: 4 μg kg^−1^ BW week^−1^; MeHg: 1.3 µg kg^−1^ BW week^−1^) [35] and with the recommended dietary value of Se (RDA: 30 μg day^−1^ in children of 4–8 years old) [36].

### 2.5. Molar Ratio (Se:Hg) and Selenium Health Benefit Value (HBV_Se_)

The molar ratio (Se:Hg) (μmol g^−1^) was calculated individually for each fish species dividing Se and Hg concentrations by their respective molecular weights (Hg: 200.59; Se: 78.96). The selenium health benefit value (HBV_Se_) was calculated using the molar concentrations of two elements according to the following equation [37]:

HBV_Se_ = [(Se − Hg)/Se] × (Se + Hg).

A positive value of HBV_Se_ is considered healthy, whereas a negative value indicates health risks associated with Hg exposure.

### 2.6. Daily and Monthly Consumption Rate Limit

The maximum allowable daily fish consumption rate (CR_lim_) (g day^−1^) for non-carcinogenic effects was computed using the following equation [38]:

CR_lim_ = (RfD × BW)/C,

where RfD is reference dose (MeHg: 1 × 10^−4^ mg kg^−1^ day^−1^) determined by the US EPA [39]; BW is consumer body weight (16 kg); and C is the measured concentration of MeHg in the edible portion of a given species of fish (μg g^−1^). The maximum allowable daily fish consumption rates (CR_lim_) were transformed to the allowable number of fish meals per month (CR_mm_) (meals/month) in accordance with the following equation:

CR_mm_ = (CR_lim_ × Tap)/MS,

where Tap is the average of exposure time (30.44 days per month), and MS is meal size (0.114 kg for children) [38]. If the number of meals of a contaminated fish species is higher than 16 per month, it indicates that there is no obvious human health risk by consumption of the fish species [38].

### 2.7. Statistical Analysis

The Kruskal–Wallis test was carried out to check whether the levels of total mercury, methylmercury, and selenium varied significantly among different fish species. The level of significance set at *p* ≤ 0.05 was adopted.

## 3. Results and Discussion

### 3.1. Concentrations of Total Mercury (THg), Methylmercury (MeHg), and Selenium (Se)

As can be seen in Table 2, THg and MeHg concentrations varied widely among the different families and species of fish investigated (THg: 0.03–0.64 μg g^−1^; MeHg: 0.02–0.55 μg g^−1^). The proportion of MeHg relative to THg, which is expressed as a percentage, ranged from a minimum of 66.7% in European anchovy to a maximum of 92.9% in Atlantic bonito, indicating that in fish muscle tissue MeHg represents the bulk of total Hg, as documented in numerous other studies [40].

**Figure 1:**
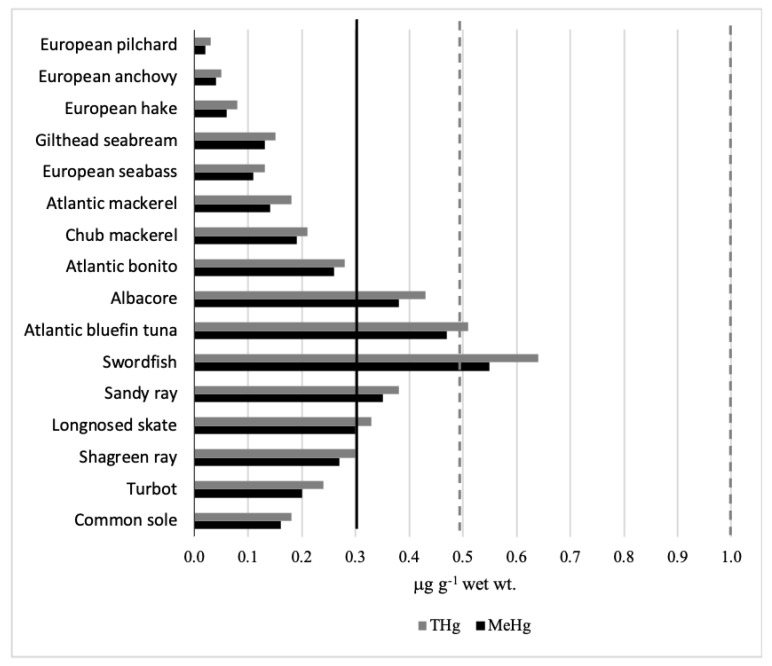
Concentrations of total mercury (THg) and methylmercury (MeHg) in fish muscle tissue in comparison to international guidelines. Dashed gray lines: maximum concentration of THg (0.5 and 1 µg g^−1^ wet wt.) [42]; black line: maximum concentration of MeHg (0.3 µg g^−1^ wet wt.) [43].

The key of interpretation for this large interspecific difference in THg and MeHg levels (~20–30 fold between the lowest and the highest value for Hg and MeHg, respectively) is in the complexity of the interactions involving biological (growth rate, size, sex, age), ecological (food, habitat), and environmental factors (Hg availability, methylation rate, primary productivity), which affect the bioaccumulative process of Hg in marine biota [41]. Furthermore, due to its great mobility in the marine ecosystem, Hg biomagnifies efficiently through the trophic chain reaching high doses in top-level predators. Consequently, as revealed by statistical analysis, pelagic carnivorous species occupying a terminal position in marine trophic pyramids such as swordfish (THg: 0.64, MeHg: 0.55 μg g^−1^), Atlantic bluefin tuna (THg: 0.51, MeHg: 0.47 μg g^−1^), and albacore (THg: 0.43, MeHg: 0.38 μg g^−1^) showed greater concentrations than the others pelagic carnivorous fish, Atlantic bonito (THg: 0.28, MeHg: 0.26 μg g^−1^), chub mackerel (THg: 0.21, MeHg: 0.19 μg g^−1^), and Atlantic mackerel (THg: 0.18, MeHg: 0.14 μg g^−1^), which are species with similar ecology (i.e., feeding traits and life history) (H = 3.86; *p* = 0.05). Concentrations statistically lower were found in gilthead seabream (THg: 0.15, MeHg: 0.13 μg g^−1^) and European seabass (THg: 0.13, MeHg: 0.11 μg g^−1^), both omnivorous species, followed by European hake (THg: 0.08, MeHg: 0.06 μg g^−1^) and by the smallest pelagic zooplanctivore species such as European anchovy (THg: 0.05, MeHg: 0.04 μg g^−1^) and European pilchard (THg: 0.03, MeHg: 0.02 μg g^−1^)(H = 5.00; *p* < 0.03). Among the benthic fishes, piscivorous species such as sandy ray (THg: 0.38, MeHg: 0.35 μg g^−1^), longnose skate (THg: 0.33, MeHg: 0.30 μg g^−1^) and shagreen ray (THg: 0.30, MeHg: 0.27 μg g^−1^) showed consistent Hg levels, whereas more moderate concentrations were in turbot (THg: 0.24, MeHg: 0.20 μg g^−1^) and common sole (THg: 0.18, MeHg: 0.16 μg g^−1^) (H = 3.00; *p* > 0.05). In general, organisms dwelling during their life in strong contact with sediments and from where they mainly feed are more readily exposed to the greater quantities of Hg that accumulate in sediments than other fish, confirming either the considerable process of sedimentation of this metal in marine depths or the importance of feeding patterns on Hg accumulation process in fish. However, independently from complex mercury dynamics in aquatic ecosystems, the studied species exhibited Hg values within the European Commission Regulation [42], which establishes Hg maximum limit in whole fresh fish at 0.50 μg g^−1^, except for predatory species for which the accepted tolerance level raises to 1 μg g^−1^. Furthermore, looking to MeHg, the most dangerous mercury compound, a more stringent guideline value (0.30 μg g^−1^) was fixed by the US EPA [43]. In this case, the fish exceeding the above-mentioned MeHg value were mostly large predators (albacore, Atlantic bluefin tuna, and swordfish) and benthic species (Raja spp.) (Figure 1).

Regarding Se, the concentrations from 0.15 µg g^−1^ to 0.58 μg g^−1^ were significantly lower than those of THg and MeHg (*p* < 0.02). Furthermore, lower overall variability of Se concentrations (~4 times between the lowest and highest values) compared to those of THg and MeHg was observed among various species, coherent with the homeostatic regulation of this essential element in the organism [37] and with its low transfer throughout the food webs [44]. Literature reflects disagreement on the accumulation of Se in the muscle tissue of marine fish. For example, Ulusoy et al. [45] found in benthic species, such as turbot (1.86 µg g^−1^ wet wt.) and red mullet (1.73 µg g^−1^ wet wt.), levels higher than those in large predators such as Atlantic bluefin tuna (1.05 µg g^−1^ wet wt.). Conversely, Olmedo et al. [18] note that predatory fish such as swordfish (0.49 µg g^−1^ wet wt.) and tuna (0.57 µg g^−1^ wet wt.) contain higher levels of Se compared to other fish species (0.004–0.35 µg g^−1^ wet wt.). Azad et al. [12] instead, measure the greater Se contents in the pelagic fish (0.53 µg g^−1^ wet wt.), followed by the demersal species (0.43 µg g^−1^ wet wt.) and benthopelagic fish group (0.30 µg g^−1^ wet wt.). In our study, the pelagic fish such as Atlantic bluefin tuna (0.58 µg g^−1^), albacore (0.52 µg g^−1^), and swordfish (0.44 µg g^−1^), had significantly higher concentrations of Se (H = 3.75; *p* = 0.05) compared either to the Scombridae family fish (Atlantic bonito: 0.44 µg g^−1^, Atlantic mackerel: 0.40 µg g^−1^, chub mackerel: 0.33 µg g^−1^) or to clupeids and in remaining pelagic species (H = 6.72; *p* < 0.01) having levels equal or below 0.30 µg g^−1^. For benthic fish, Se concentrations ranged from 0.28 µg g^−1^ up to 0.47 µg g^−1^ with a greater enrichment in the different elasmobranch species (sandy ray: 0.47 µg g^−1^, shagreen ray: 0.44 μg^−1^, longnose skate: 0.41 µg g^−1^) with respect to the other two considered fishes (turbot: 0.30 µg g^−1^, common sole: 0.28 µg g^−1^). However, despite the fluctuation of Se content, the statistical analysis did not highlight any significant differences in concentrations between the considered fish categories (H = 3.00; *p* > 0.05).

### 3.2. Exposure Assessment and Dietary Reference Intake

The estimated THg and MeHg exposures from fish consumption by children are described in Table 2. A wealth of scientific literature describes the adverse neurological effects caused by exposure to Hg. The fetus and young children are more vulnerable than adults to the risk of neurologic alterations due to greater sensitivity during the early stages of brain development [46]. In this scenario, the European Food Safety Agency (EFSA) and the Food and Drug Administration (FDA) have advised vulnerable population groups, such as pregnant women and young children, to avoid the consumption of some types of fish, especially large predators, prone to accumulate Hg high levels [47]. However, both vulnerable classes and fish consumers of all ages and genders can be at risk of Hg contamination. Consequently, provisional tolerable weekly intakes (PTWIs) of 4 µg kg^−1^ BW week^−1^ and of 1.3 µg kg^−1^ BW week^−1^ for THg and MeHg, respectively, have been established by EFSA [35] as the amount of a substance that can be consumed weekly over an entire lifetime without any significant risk to human health. As can be seen in Table 2, the highest THg exposure levels, exceeding the safe dose, were due to the consumption of large predators with swordfish in a key position (6.36 µg kg^−1^ BW week^−1^), followed by Atlantic bluefin tuna (5.07 µg kg^−1^ BW week^−1^) and albacore (4.27 µg kg^−1^ BW week^−1^). Moreover, the consumption of Atlantic mackerel (1.79 µg kg^−1^ BW week^−1^), chub mackerel (2.09 µg kg^−1^ BW week^−1^), Atlantic bonito (2.78 µg kg^−1^ BW week^−1^), and of the benthic species (1.79–3.78 µg kg^−1^ BW week^−1^) determined an exposure rather high but within the safe level, while eating fish as gilthead seabream (1.49 µg kg^−1^ BW week^−1^), European seabass (1.29 µg kg^−1^ BW week^−1^), European hake (0.80 µg kg^−1^ BW week^−1^) or anchovy (0.50 µg kg^−1^ BW week^−1^) and sardine (0.30 µg kg^−1^ BW week^−1^) led to low or moderate Hg intakes. The scenario was different for MeHg because the weekly intakes were close (gilthead seabream: 1.29 µg kg^−1^ BW week^−1^) or higher than MeHg PTWI in all cases (1.39–5.47 µg kg^−1^ BW week^−1^), except for consumption of European seabass: 1.09 µg kg^−1^ BW week^−1^, European hake: 0.60 µg kg^−1^ BW week^−1^, and clupeids: 0.20–0.40 µg kg^−1^ BW week^−1^, which were within the safe level. With respect to Se, a micronutrient of fundamental importance for many bodily processes, an adequate intake via diet for all age groups is desirable. However, as with all essential elements, low or moderate Se intakes are necessary to sustain life but excessive intakes can produce toxicity. A dietary Se excess can result in selenosis whose symptoms include gastrointestinal upsets, hair loss, white blotchy nails, fatigue, and irritability [48], while a severe Se deficiency is associated with Keshan disease, in which cardiomyopathy occurs mainly during preadolescent or adolescent years [49]. For this essential micronutrient, a provisional tolerable intake does not exist. However, the estimated daily intakes (EDI) varying from 0.21 µg kg^−1^ BW day^−1^ to 0.82 µg kg^−1^ BW day^−1^ provided from 0.71% to 2.75% of the recommended dietary allowance (RDA) [36], with Atlantic bluefin tuna representing the major contributor to Se intake.

### 3.3. Selenium:Mercury Molar Ratio

**Figure 2:**
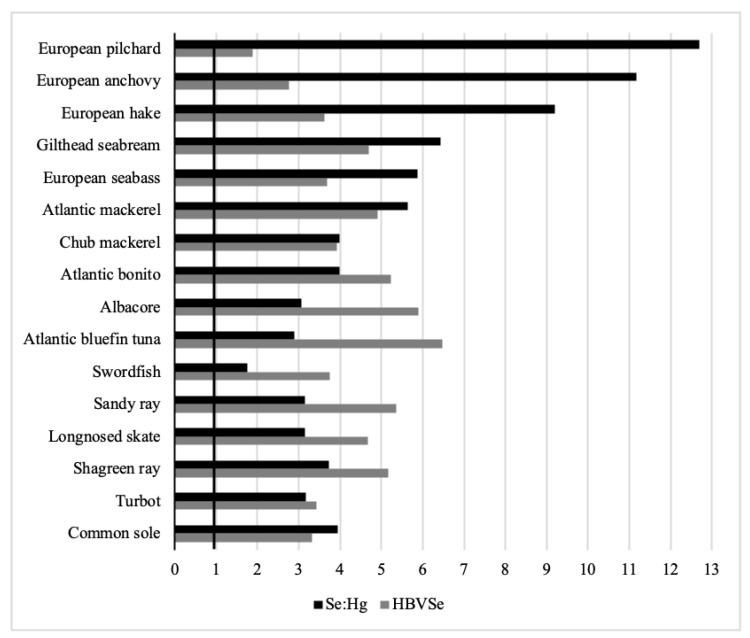
Se:Hg molar ratios and Se health benefit values (HBV_Se_) of the studied fish species. Dark line: Se:Hg molar ratio > 1.

Dietary Se intake may determine a positive effect on the toxicological outcomes of Hg exposure; contemporarily, estimates of Se intake alone may not adequately reflect the health risk/benefit of Se if its relationship with Hg is not carefully pondered. A lot of studies have, in fact, confirmed that Se not only moderates the uptake of Hg but counteracts its toxicity in a multitude of animal species, including fish and humans [50,51]. The exact mechanisms are currently not fully defined, but most of them involve the formation of Hg–Se compounds [9,52], which are rarely bioavailable and facilitate the removal and excretion of MeHg by demethylation [53]. Consequently, when examining the health problems associated with exposure to Hg, especially from consumption of fish representing the main path for human exposure to Hg, is a crucial priority to examine the interactions between these two elements. [37]. In general, a surplus of Se with respect to Hg provides a potential shield from negative Hg consequences. Specifically, when Se:Hg ratio, computed as a molar ratio, exceeded 1, the Se protective effect against Hg toxicity occurs [50]. In our case, as graphically illustrated in Figure 2, it appeared that Se:Hg molar ratios, although differing among species, were all greater than 1. In particular, the highest Se:Hg molar ratio occurred in clupeids (anchovy: 11.18; sardine: 12.70) and in gadoid European hake (9.21), and intermediate values were found in the other pelagic species, including gilthead seabream (6.44), seabass (5.86), Atlantic mackerel (5.65), chub mackerel (3.99), Atlantic bonito (3.99) and albacore (3.07), while the lowest were measured in two carnivores of more large size as Atlantic bluefin tuna (2.89) and swordfish (1.75).

Within deep-sea fish, the Se:Hg molar ratio variation was moderately large, with the common sole (3.95) and shagreen ray (3.73) exhibiting the highest values, followed by the remaining fish species having similar molar ratios (3.14–3.16). It is clear that the observed decrease in values of Se:Hg molar ratio as it moves along the food chain from planktivorous to omnivorous and carnivorous species is dependent on a combination of several factors (e.g., fish size, season, and sampling location) [17] but very more logically reflects the substantial differences in Hg and Se concentrations due to differences in the processes of bioaccumulation and trophic transfer existing between two elements. Our data, in fact, fit well into a general picture showing that Se:Hg molar ratio is higher in species occupying a low position in the trophic pyramids and decreases with increasing trophic level and organism age and size [11,19]. This trend indicates that top-level predators may not offer the best Se protection against Hg toxicity for consumers, in comparison to other fish. In this respect, our results showed that swordfish was the only analyzed species that presented a Se:Hg ratio nearly equimolar, suggesting the impossibility of Se to totally balance the potentially toxic effects of Hg. However, the variability of Se:Hg molar ratio found within and across species diminishes its usefulness for establishing food safety considerations [54]. An innovative parameter, the selenium health benefit value (HBV_Se_) provides a more trustworthy key to evaluate Hg exposure risks [37].

Analysis of our data in terms of HBV_Se_ showed a moderate inter-species variation with the lowest values in clupeids (sardine: 1.89, anchovy: 2.76) and the greatest in Atlantic bluefin tuna (6.47), albacore (5.89), Atlantic bonito (5.22), and in the elasmobranch group (4.67–5.35), while in the remaining species, including the common sole, turbot, European hake, European seabass, chub mackerel, and swordfish, the values ranged from 3.32 to 3.92 (Figure 2). Looking to these findings with all Se:Hg molar ratios higher than 1 and the HBV_Se_ index positive, the consumption of all studied species could be deemed safe. However, because it is a public health issue, Ralston et al. (2016) suggest that a precautionary principle must prevail even when the HBV_Se_ index is positive. This assumption becomes more realistic especially when measuring positive but low HBV_Se_ values. In our study, modest values of this index have been calculated for all species, but especially for sardine (1.89), revealing the possibility of a potential health hazard as a result of their consumption. The obtained data corroborate previous studies reporting beneficial HBV_Se_ and favorable Se:Hg ratios in almost all seafood, except top predators for which contrasting findings were observed. In sharks, for example, Kaneko and Ralston [55] and Olmedo et al. [18] presented Se–HBV and Hg:Se molar ratios both negative, in contrast with other authors reporting molar ratios higher than 1 and HBV_Se_ index either negative [16] or positive [13]. In swordfish, Calatayud et al. [56] measured a negative Se–HBV value, whereas Cabañero et al. [51] and Olmedo et al. [18] found favorable Se:Hg molar ratios very close to our results. For tuna, these contradictions do not emerge but exists a consensus general indicating a healthy profile. All literature data, in fact, reflect positive HBV_Se_ or Se–HBV and healthy molar ratios [18,57,58,59], showing that consumption of this species is considered safe in terms of Hg exposure risks for consumers.

### 3.4. Daily and Monthly Consumption Rate Limit

**Figure 3:**
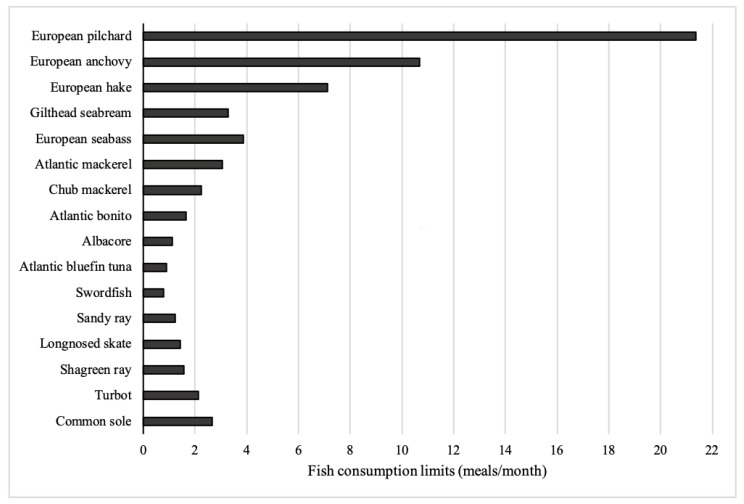
Maximum allowable fish consumption rate in meals/month (CR_mm_) for children without adverse health effects.

With regard to Hg, establishing restrictions on the consumption of fish and other aquatic species for the general population, but especially for sensitive sub-groups of the population such as pregnant women and children, allows to reduce exposure and, at the same time, to reap the nutritional benefits provided by this food. To protect and help these most vulnerable population groups to make informed choices about the type of fish to consume, the United States Food and Drug Administration and the United States Environmental Protection Agency [60] have created an easy-to-use reference chart that classifies fish as “Best choices” “Good choices” and mostly large predatory fish, such as shark, swordfish, and king mackerel, the consumption of which should be avoided because they are particularly rich in Hg. In this context, an important aspect of the assessment of risks to human health is the estimation of the quantity of fish that can be securely ingested over a given time period with no adverse effects. This information given in terms of the maximum allowable monthly consumption limits (CR_mm_) is shown in Figure 3 (Supplemental Table S1). More specifically, children may safely consume 21 meals of sardine per month with no adverse non-carcinogenic health effects, 11 and 7 meals of European anchovy and European hake, respectively, but not more than 1, 2, or 3 meals in a month of large predators and benthic species.

### 3.5. Uncertainties and Limitations

The analysis of uncertainty is a usual component of risk assessment because there are many variables, including per capita consumption, consumption frequency, metal concentration, consumer body weight, exposure time period, etc. that can greatly affect the interpretation of results. Consequently, when discussing human exposure, uncertainties and limitations need to be acknowledged. In our case, more accurate information on the consumption of each individual fish by children and body weight at each age are necessary parameters to reduce the uncertainty associated with exposure. There are limitations even when using HBV_Se_ as a tool of health risk assessment because the studies on the interactions between Se and Hg involve non-human models and consequently the exact molar ratio at which neutralizing effects of Se over MeHg occurs in human is unknown [61]. Moreover, the culinary treatments leading to change of essential/toxic element bioaccessibility can contribute to increasing the uncertainty of estimated exposure levels [62]. However, the principal uncertainty in our dataset arises from the lack of information about the size of swordfish, Atlantic bluefin tuna, and albacore because it is well known that there is a direct link between fish size and the presence of mercury, especially in top predatory fish [63,64].

## 4. Conclusions

There is wide scientific evidence supporting the benefits and potential harms of consuming fish, and this problem becomes even more important when it involves harmful chemicals such as mercury and high-risk groups such as children for whom various factors (immature chemical detox systems, rapid growth) contribute to creating critical windows of vulnerability that can determine lifetime consequences [65]. Information regarding Hg and MeHg levels, Se, and Se:Hg molar ratios in commercial marine fish are, therefore, crucial and should always be taken into account for the safety of marine product consumption. On this basis, our results indicate that, even if the Hg and MeHg intake exceed the recommended standards, Se content in fish could be sufficient to mitigate the potentially toxic effects resulting from exposure to this toxin. However, as substantial uncertainty still exists in understanding the relationships between Se and Hg and human health, the results should be interpreted with prudence. This is particularly true for species such as swordfish and Atlantic bluefin tuna, which show low or almost equimolar Se:Hg ratios, and their HBV_Se_ is not particularly high. This precautionary principle must predominate also in the light of the results relative to the maximum allowable fish consumption rates. The application of this parameter linked to non-carcinogenic health effects suggests that monthly consumption of swordfish, Atlantic bluefin tuna, albacore, and Atlantic bonito should be extremely moderate and this advice should be extended to these high-trophic level pelagic species and to other specific species (see sandy ray and longnose skate) in consideration of the low safe consumption frequency calculated. These results highlight the importance to develop guidelines on the amounts, types, and frequency of fish consumption that currently do not exist in Italy, unlike other countries where bans, advice, and recommendations for vulnerable population groups, such as young children and women during the reproductive period, have already been in vogue for some time.

## Figures and Tables

**Table 1:** Nomenclature, total length (min-max and mean ± SD) and trophic levels of the studied species.

Scientific Name ^1^	Common Name	n° Specimens	Length	**Trophic Level**
Demersal-pelagic fish				
*Sardina pilchardus*	European pilchard	20	14.6–20.8 17.7 ± 1.9	2.76 ^2^
*Engraulis encrasicolus*	European anchovy	20	12.0–20.0 16.1 ± 2.5	3.10 ^2^
*Merluccius merluccius*	European hake	15	21.5–30.8 25.0 ± 3.2	4.09 ^2^
*Sparus aurata*	Gilthead seabream	10	29.3–35.4 31.9 ± 2.3	3.42 ^2^
*Dicentrarchus labrax*	European seabass	10	26.0–37.0 32.0 ± 3.9	3–4.6 ^3^
*Scomber scombrus*	Atlantic mackerel	10	25.0–36.7 30.4 ± 3.9	4.14 ^2^
*Scomber japonicus*	Chub mackerel	15	12.5–26.8 19.0 ± 4.9	3.99 ^3^
*Sarda sarda*	Atlantic bonito	8	45.0–56.3 50.0 ± 3.7	4.48 ^2^
*Thunnus alalunga*	Albacore	15 *	-	4.47 ^4^
*Thunnus thynnus*	Atlantic bluefin tuna	15 *	-	4.30 ^2^
*Xiphias gladius*	Swordfish	15 *	-	4.46 ^2^
Benthic fish				
*Leucoraja circularis*	Sandy ray	8	40.0–56.5 47.9 ± 5.8	3.80 ^5^
*Dipturus oxyrinchus*	Longnosed skate	8	55.8–70.2 61.4 ± 5.9	3.75 ^2^
*Leucoraja fullonica*	Shagreen ray	8	43.7–58.8 49.5 ± 5.6	3.80 ^5^
*Scophthalmus maximus*	Turbot	10	35.0–48.0 39.0 ± 4.3	4.4 ^6^
*Solea solea*	Common sole	10	20.0–38.0 26.1 ± 5.5	2.95 ^2^

^1^ [26]; ^2^ [27]; ^3^ [28]; ^4^ [29]; ^5^ [30]; ^6^ [31]. * For these species were analyzed slices.

**Table 2:** Concentrations (µg g^−1^ wet weight) and estimated intakes (EWI: µg kg^−1^ BW week^−1^; EDI: µg kg^−1^ BW day^−1^) of total mercury (THg), methylmercury (MeHg), and selenium (Se).

Species	THg	MeHg	Se	THg EWI	MeHg EWI	Se EDI
Pelagic fish						
European pilchard	0.03	0.02	0.15	0.30	0.20	0.21
European anchovy	0.05	0.04	0.22	0.50	0.40	0.31
European hake	0.08	0.06	0.29	0.80	0.60	0.41
Gilthead seabream	0.15	0.13	0.38	1.49	1.29	0.54
European seabass	0.13	0.11	0.30	1.29	1.09	0.43
Atlantic mackerel	0.18	0.14	0.40	1.79	**1.39**	0.57
Chub mackerel	0.21	0.19	0.33	2.09	**1.89**	0.47
Atlantic bonito	0.28	0.26	0.44	2.78	**2.58**	0.62
Albacore	0.43	0.38	0.52	**4.27**	**3.78**	0.74
Atlantic bluefin tuna	0.51	0.47	0.58	**5.07**	**4.67**	0.82
Swordfish	0.64	0.55	0.44	**6.36**	**5.47**	0.62
Benthic fish						
Sandy ray	0.38	0.35	0.47	3.78	**3.48**	0.67
Longnose skate	0.33	0.30	0.41	3.28	**2.98**	0.58
Shagreen ray	0.30	0.27	0.44	2.98	**2.68**	0.62
Turbot	0.24	0.20	0.30	2.39	**1.99**	0.43
Common sole	0.18	0.16	0.28	1.79	**1.59**	0.40

In bold values of EWI > 4 µg kg^−1^ b.w. (THg). EWI > 1.3 µg kg^−1^ b.w. (MeHg).

## Supplementary Materials

The following are available online at https://www.mdpi.com/2305-6304/9/2/39/s1, Table S1: THg and MeHg concentrations and daily (CRlim) and monthly (CRmm) consumption rate limit in children.
