# Supplementary material for: Levels of Mercury, Methylmercury and Selenium in Fish: Insights into Children Food Safety
Source: Toxics. 2021 Feb 20;9(2):39. doi: 10.3390/toxics9020039 (PMC7923435; doi:10.3390/toxics9020039)
Supplement: Supplementary file 1 [file toxics-09-00039-s001.pdf]

Article

Supplementary Materials: Levels of Mercury, Methylmercury and Selenium in Fish: Insights into Children Food Safety

Grazia Barone, Arianna Storelli, Daniela Meleleo, Angela Dambrosio, Rita Garofalo, Antonio Busco and Maria Maddalena Storelli

Table S1. THg and MeHg concentrations and daily (CR_{lim}) and monthly (CR_{mm}) consumption rate limit in children.

Species	THg			MeHg		
	μg g⁻¹ w.w.	CR_{lim} (g d⁻¹)	CR_{mm} (meals/month)	μg g⁻¹ w.w.	CR_{lim} (g d⁻¹)	CR_{mm} (meals/month)
European anchovy	0.03	87	23	0.02	131	35
European pilchard	0.05	52	14	0.04	66	18
European hake	0.08	33	9	0.06	44	12
Gilthead seabream	0.15	17	5	0.13	20	5
European seabass	0.13	20	5	0.11	24	6
Atlantic mackerel	0.18	15	4	0.14	19	5
Chub mackerel	0.21	12	3	0.19	14	4
Atlantic bonito	0.28	9	3	0.26	10	3
Albacore	0.43	6	2	0.38	7	2
Atlantic bluefin tuna	0.51	5	1	0.47	6	1
Swordfish	0.64	4	1	0.55	5	1
Sandy ray	0.38	7	2	0.35	7	2
Longnosed skate	0.33	8	2	0.30	9	2
Shagreen ray	0.30	9	2	0.27	10	3
Turbot	0.24	11	3	0.20	13	4
Common sole	0.18	15	4	0.16	16	4
European anchovy	0.03	87	23	0.02	131	35
European pilchard	0.05	52	14	0.04	66	18
European hake	0.08	33	9	0.06	44	12
Gilthead seabream	0.15	17	5	0.13	20	5
European seabass	0.13	20	5	0.11	24	6
Atlantic mackerel	0.18	15	4	0.14	19	5
Chub mackerel	0.21	12	3	0.19	14	4
